# P‐cresyl sulfate causes mitochondrial hyperfusion in H9C2 cardiomyoblasts

**DOI:** 10.1111/jcmm.15303

**Published:** 2020-07-08

**Authors:** Tien‐Hung Huang, Hon‐Kan Yip, Cheuk‐Kwan Sun, Yi‐Ling Chen, Chih‐Chao Yang, Fan‐Yen Lee

**Affiliations:** ^1^ Division of Cardiology Department of Internal Medicine Kaohsiung Chang Gung Memorial Hospital and Chang Gung University College of Medicine Kaohsiung Taiwan; ^2^ Institute for Translational Research in Biomedicine Kaohsiung Chang Gung Memorial Hospital and Chang Gung University College of Medicine Kaohsiung Taiwan; ^3^ Center for Shockwave Medicine and Tissue Engineering Kaohsiung Chang Gung Memorial Hospital and Chang Gung University College of Medicine Kaohsiung Taiwan; ^4^ Department of Medical Research China Medical University Hospital China Medical University Taichung Taiwan; ^5^ Department of Nursing Asia University Taichung Taiwan; ^6^ Department of Emergency Medicine E‐Da Hospital I‐Shou University School of Medicine for International Students Kaohsiung Taiwan; ^7^ Division of Nephrology Department of Internal Medicine Kaohsiung Chang Gung Memorial Hospital and Chang Gung University College of Medicine Kaohsiung Taiwan; ^8^ Division of Thoracic and Cardiovascular Surgery Department of Surgery Kaohsiung Chang Gung Memorial Hospital and Chang Gung University College of Medicine Kaohsiung Taiwan; ^9^ Division of Cardiovascular Surgery Department of Surgery Tri‐Service General Hospital National Defense Medical Center Kaohsiung Taiwan

**Keywords:** mitochondria, p‐cresyl sulphate, stress‐induced mitochondrial hyperfusion

## Abstract

Increased circulating level of uraemic solute p‐cresyl sulphate (PCS) in patients with chronic kidney disease (CKD) is known to increase myocardial burden relevant to mitochondrial abnormalities. This study aimed at investigating mitochondrial response to PCS in H9C2 cardiomyoblasts. H9C2 cardiomyoblasts were treated with four different concentrations of PCS (3.125, 6.25, 12.5 and 25.0 µg/mL) to study the changes in cell proliferation, cell size and mitochondrial parameters including morphology, respiration, biogenesis and membrane potential. The lowest effective dose of PCS (6.25 µg/mL) induced mitochondrial hyperfusion with enhanced mitochondrial connectivity, mitochondrial oxygen consumption rates, mitochondrial mass, mitochondrial DNA copy number and increased volume of cardiomyoblasts. After PCS treatment, phosphorylation of energy‐sensing adenosine monophosphate‐activated protein kinase (AMPK) was increased without induction of apoptosis. In contrast, mitochondrial mass was recovered after AMPK silencing. Additionally, mitochondrial hyperfusion and cell volume were reversed after cessation of PCS treatment. The results of the present study showed that low‐level PCS not only caused AMPK‐dependent mitochondrial hyperfusion but also induced cell enlargement in H9C2 cardiomyoblasts in vitro.

## 1 INTRODUCTION

The risk of premature death is fivefold to tenfold higher in patients with chronic kidney disease (CKD) compared with that of their progressing to end‐stage kidney disease. Cardiovascular disease is found to be the major contributor to the exponentially increased risk of death as kidney function deteriorates.^1^ One of the significant problems in patients with CKD is the accumulation of different toxic chemicals that cause uraemic symptoms and cardiac remodelling.^2^ Uraemic solute p‐cresyl sulphate (PCS) is a metabolized product originating from intestinal microbial fermentation and difficult to be removed through conventional haemodialysis.^3^ Several studies have shown that PCS is able to induce oxidative stress in endothelial cells, vascular smooth muscle cells, renal tubular cells and cardiomyocytes.^4^, ^5^, ^6^ Additionally, a recent study has reported that increased serum total PCS levels are associated with a worse prognosis in stable angina patients with early‐stage renal failure.^7^

Mitochondria are energy‐generating organelles, the functions of which are intrinsically linked to their morphology and membrane structure. Mitochondria stay in a dynamic balance through switching between mitochondrial fusion and fission for maintaining a physiological cellular mitochondrial population.^8^ Accumulated evidence has demonstrated that progression of cardiovascular diseases is accompanied by increased mitochondrial damage and dysfunction.^9^ A previous study has demonstrated that mitochondrial hyperfusion with increased mitochondrial ATP production can be maintained as long as the cells are exposed to some specific stress stimuli. This phenomenon has been known as 'stress‐induced mitochondrial hyperfusion' (SIMH).^10^The stress stimuli reported to trigger mitochondrial hyperfusion as a protective response against stress include starvation, UV irradiation and actinomycin D.^10^ However, little is known about the mitochondrial defence mechanism against potential threat under mild uraemic conditions. To mimic the clinical scenario of CKD at different stages of development, the present study attempted to address the effects of increasing concentrations of PCS on mitochondrial functions in cardiomyoblasts.

## 2 MATERIALS AND METHODS

### 2.1 Cell culture

H9C2 cardiomyoblasts were purchased from Bioresource Collection and Research Center (BCRC) and were maintained in a proliferative state (ie undifferentiated condition) by cultured with DMEM (high glucose) medium (11965‐084, GIBCO) at 37°C under 5% CO2. P‐cresyl sulphate (PCS) was purchased from Apexbio (A8895). During PCS treatment, different concentrations of PCS were added in fresh medium every three days.

### 2.2 Assessment of cell proliferation by Ki67 staining and CCK8 assay

For Ki67 staining, H9C2 cardiomyoblasts were fixed with cold 4% paraformaldehyde followed by permeabilization with 0.1% Triton X‐100. Fixed cells were incubated with murine anti‐Ki67 monoclonal antibody (1:500, ab155801, Abcam) and goat anti‐rabbit Alexa Fluor 594 secondary antibody, as well as counterstained with DAPI. The cells were examined by fluorescence microscopy. For CCK8 assay, H9C2 cardiomyoblasts growing in 96‐well culture dish were treated with PCS in different concentrations for three days. At the end of the culture, 10 μL of the CCK8 reagent (96992, SIGMA‐ALDRICH) was added to each well. After two hours of incubation at 37°C, the absorbance was determined by spectrophotometer at 450 nm.

### 2.3 Determination of mitochondrial network complexity

Mitochondria in H9C2 cardiomyoblasts were labelled with MitoTracker Orange (M7510, Thermo Fisher Scientific) by incubating the cells with 300 nM dye for 45 min, followed by fixing with 4% paraformaldehyde for observation. Fluorescence‐labelled mitochondria were observed by Olympus Fluoview FV10i‐DOC laser confocal microscope (Olympus). For image quantification, we collected images of more than 56 cells for each PCS concentration. Mitochondrial network complexity was analysed using Mitochondrial Network Analysis (MiNA) toolset for parameters including mean branch length, number of networks and area of mitochondrial footprints.^11^

### 2.4 Quantification of mitochondrial DNA copy

Total DNA was extracted using DNeasy Blood & Tissue kit (69504, Qiagen) following to the manufacturer's instruction. The number of mitochondrial DNA copies (ND1‐mtDNA, mitochondria‐specific DNA) in H9C2 cells was quantified by real‐time qPCR using QuantiNOVA SYBR Green PCR assay (208054, Qiagen) and normalized by rat genomic DNA (GAPDH‐DNA, intronic DNA). Triplicate assays were performed on Step One‐Plus (Applied Biosystems). Primer sequences were listed below: ND1‐mtDNA forward: 5’‐CTCCCTATTCGGAGCCCTAC‐3’; ND1‐mtDNA reverse: 5’‐ATTTGTTTCTGCTAGGGTTG‐3’ ^12^; GAPDH‐DNA forward: 5’‐ GTTACCAGGGCTGCCTTCTC −3’; GAPDH‐DNA reverse: 5’‐ GGGTTTCCCGTTGATGACC −3’; ANP forward: 5’‐ CTGCTAGACCACCTGGAGGA‐3’; ANP reverse: 5’‐ AAGCTGTTGCAGCCTAGTCC‐3’.^13^

### 2.5 Measurement of mitochondrial oxygen consumption rate (OCR)

Mitochondrial bioenergetics in H9C2 cardiomyoblasts were determined by an Extracellular Flux Analyzer (XFe24 Seahorse Bioscience) through assessing the mitochondrial oxygen consumption, basal respiration, maximal respiration, ATP production and spare respiratory capacity. Briefly, H9C2 cells (10^4^ cells/per well) were seeded in FBS‐free and sodium bicarbonate‐free DMEM (high glucose) medium. Totally 100 µM Oligomycin, 100 µM FCCP and 540 µL Antimycin A/Rotenone were added sequentially during the reactions. OCR in reactions was sequentially measured.

### 2.6 Analysis of mitochondrial membrane potential (ΔΨm)

For the analysis of ΔΨm in H9C2 cardiomyoblasts, the membrane‐permeant JC‐1 dye (Thermo Fisher Scientific) was applied according to the user manual. Although ΔΨm is high, JC‐1 forms J‐aggregates emitting red fluorescence. When ΔΨm is low, JC‐1 monomer produces green fluorescence.^14^ Totally 2 µM JC‐1 was added and incubated at 37°C under 5% CO_2_ for 30 min. JC‐1 stained H9C2 cells were subjected to FACS analysis immediately for the changes in ΔΨm. Treatment of H9C2 cardiomyoblasts with carbonyl cyanide m‐chlorophenyl hydrazone (CCCP) was used as positive control.

### 2.7 Determination of mitochondrial mass

Mitochondria in live H9C2 cardiomyoblasts were stained with fluorescence dye using 150 nM MitoTracker Green (Thermo Fisher Scientific) for 30 min. Fluorescence‐labelled mitochondria in cells were subjected to FACS analysis for the quantification of fluorescence intensity.^15^, ^16^

### 2.8 Western blot

Protein was extracted from cultured cells using cell scraper and RIPA buffer with protease and phosphatase inhibitor. Approximate amounts of protein were subjected to SDS‐PAGE and transferred to membranes. The membranes were incubated with the primary antibodies, including actin (1:6000, MAB1501, Millipore), Drp1 (1:1000, ab56788, Abcam), phospho‐Drp1 (S616) (1:1000, 3455, Cell Signaling), AMPK (1:1000, 2532, Cell Signaling), phospho‐AMPK (Thr172) (1:500, 2535, Cell Signaling), Bax (1:1000, ab32503, Abcam) and PARP (1:1000, 9542, Cell Signaling). Horseradish peroxidase (HPR)‐conjugated secondary antibody was used for binding the primary antibody. Binding bands were visualized by enhanced chemiluminescence (WBLUF0500, Millipore) and quantified using ImageJ software.

### 2.9 Transfection of siRNA

AMPK siRNA was purchased from Sigma‐Aldrich, whereas negative control siRNA was purchased from Qiagen (1027281, Qiagen). The siRNA sequences list as follows: AMPK siRNA, 5’‐CUUAUUGGAUUUCCGAAGU‐3’. Transfection of siRNA (50 nmol/L) into H9C2 cardiomyoblasts was accomplished with TransIT‐X2 dynamic delivery system (MIR6000, Mirus), according to the manufacture's recommendation. Transfected cells were then treated with PCS for further experiments.

### 2.10 Statistical analysis

GraphPad Prism software (ver. 6) (GraphPad software) was used for statistical analyses and data plotting. Data are expressed as mean ± SD. The difference in means between two groups was evaluated using the *t* test. One‐way ANOVA was used to compare multiple groups. Test for the linear trend between groups was calculated from left to right group order. *P* values of <.05 were considered statistically significant.

## 3 RESULTS

### 3.1 PCS‐induced mitochondrial hyperfusion

**Figure 1:**
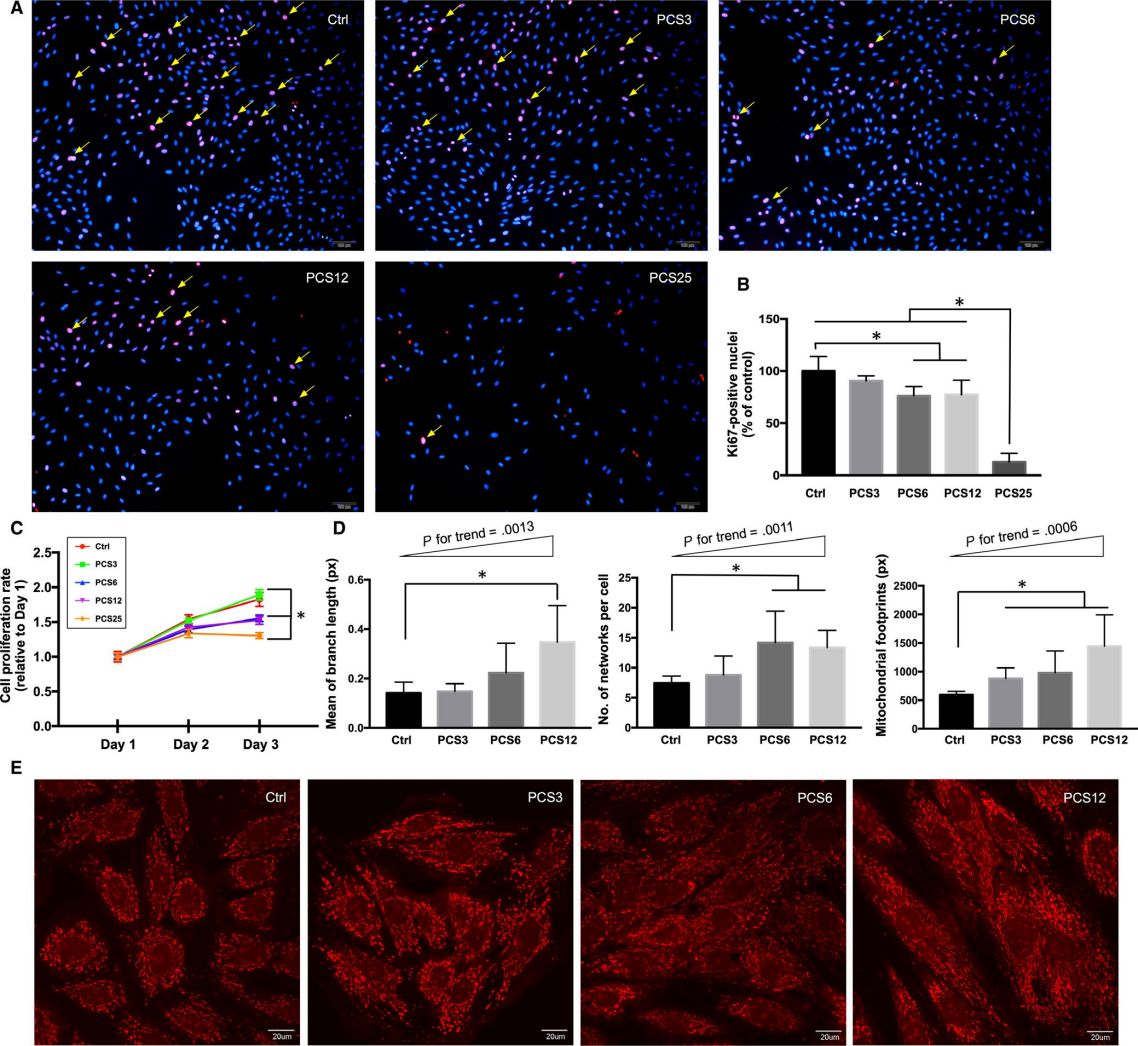
Changes in cell proliferation and mitochondrial morphology after PCS treatment. (A) Vehicle control (Ctrl, n = 5) with DMSO only and four different PCS concentrations (n = 5 for each concentration), 3.125 µg/mL (PCS3), 6.25 µg/mL (PCS6), 12.5 µg/mL (PCS12) and 25 µg/mL (PCS25) applied in H9C2 cardiomyoblasts for the assessment of proliferative potential using Ki67 staining on Day 3 after treatment. Ki67 (pink‐red) nuclei stain with DAPI (blue) in H9C2 cardiomyoblasts. (B) Quantification of Ki67 nuclei staining in different groups. (C) Determination of cell proliferation rate using CCK8 assay on Day 1, Day 2 and Day 3 after treatment. (D) After three days of PCS treatment, parameters of mitochondrial morphology including branch length, network number and footprint in Ctrl, PCS3, PCS6 and PCS12 groups, using MiNA macro tools in ImageJ software. (E) Representative morphologies of mitochondria after mitotracker orange staining (displayed in red). Each error bar represents the mean with SD, *P < .05, and px indicates pixel

To investigate the effect of p‐cresyl sulphate (PCS) on H9C2 cardiomyoblast proliferation, the cells were treated with DMSO (ie vehicle control) and four different concentrations of PCS (ie 3.125, 6.25, 12.5 and 25.0 µg/mL) that were categorized into five groups: Ctrl, PCS3, PCS6, PCS12 and PCS25, respectively. We found that cell proliferative potential was significantly lower in groups of PCS6, PCS12 and PCS25 than that in group of Ctrl through the assessments of Ki67 staining and CCK8 assay (Figure 1A‐C; *P* < .05). To evaluate whether PCS triggered abnormal mitochondrial dynamics in a dose‐dependent manner, the same grouping was applied in H9C2 cells for the comparisons of mitochondrial morphology. Among these groups, PCS25 group was removed because of the lowest cell proliferation rate after treatment. By comparing vehicle control to PCS‐treated H9C2 cells, there were significant progressive increases in the structural complexities of mitochondria with increasing PCS concentrations as reflected by the mitochondrial parameters of branch length (*P* for trend = .0013), network number (*P* for trend = .0011) and footprint area (*P* for trend = .0006) (Figure 1D,E). Both PCS6 and PCS12 groups displayed significantly higher network number and larger footprint area compared to those in the Ctrl group (*P* < .05). Also, PCS12 group showed a significantly longer branch length than that of Ctrl group (*P* < .05).

### 3.2 PCS‐triggered changes in mitochondrial bioenergetics

**Figure 2:**
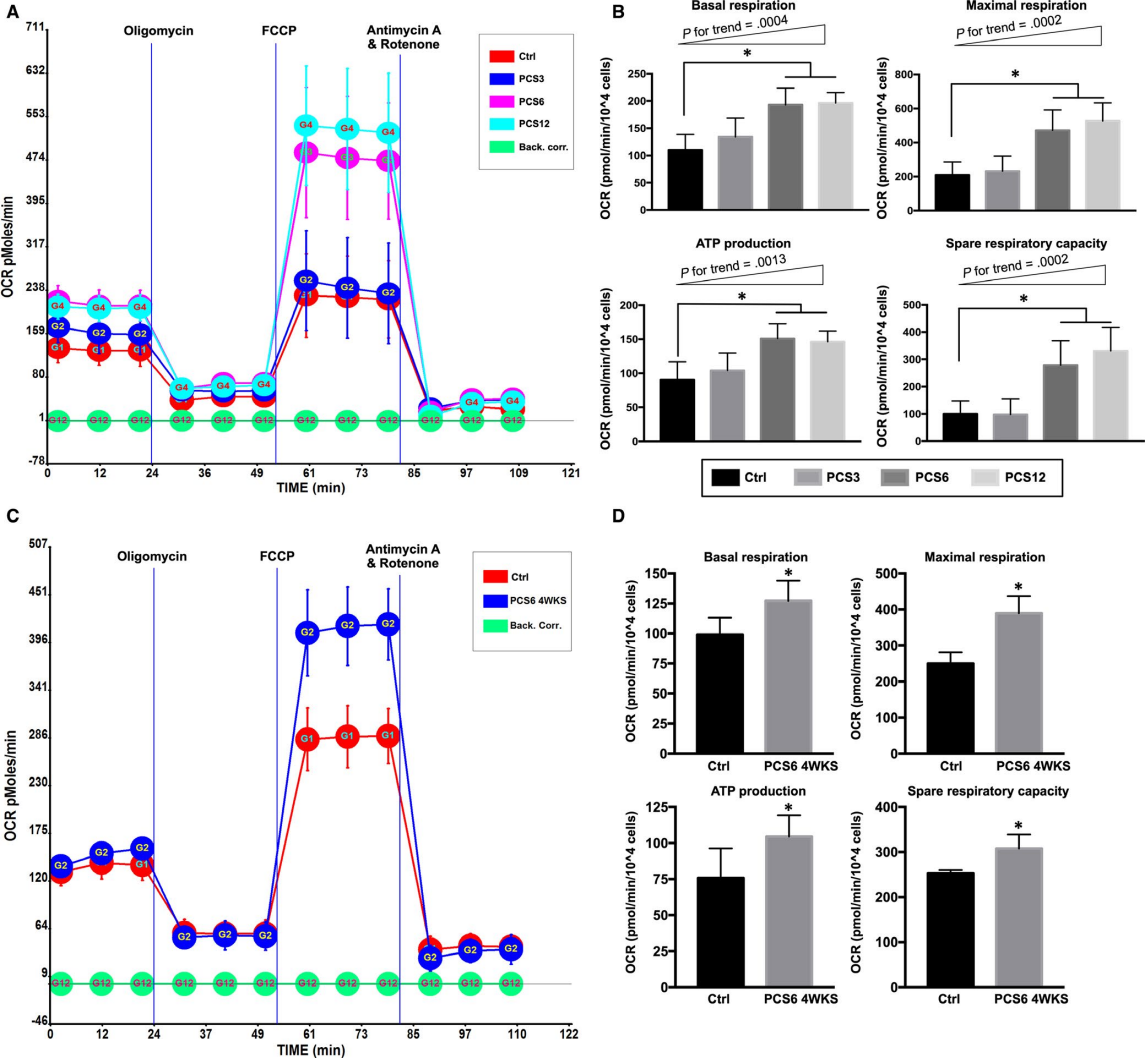
Changes in mitochondrial respiration of H9C2 cells after PCS treatment. (A) After three days of PCS treatment, mitochondrial respiration reflected by the level of oxygen consumption rate (OCR) in Ctrl, PCS3, PCS6 and PCS12 groups (n = 4 per group), following the injection of oligomycin, FCCP and antimycin A/rotenone (Back. corr.= Background correction). (B) The rates of basal respiration, maximal respiration, ATP production and spare respiratory capacity (n = 4 per group). (C) After four weeks of PCS treatment, mitochondrial respiration in Ctrl and PCS6 groups (n = 7 per group). (D) Parameters of mitochondrial respiration (n = 7 per group). Each error bar represents the mean with SD, *P < .05

To estimate whether PCS impaired mitochondrial respiration, groups of Ctrl, PCS3, PCS6 and PCS12 were subjected to Seahorse XF24 extracellular flux analyser for assessing efficiency of mitochondrial respiration reflected by the level of oxygen consumption rate (OCR) (Figure 2A). By comparing the vehicle control with the PCS‐treated groups, there were significant progressive increases in the parameters of mitochondrial respiration, including basal respiration (*P* for trend = .0004), maximal respiration (*P* for trend = .0002), ATP production (*P* for trend = .0013) and spare respiratory capacity (*P* for trend = .0002) (Figure 2B). Both PCS6 and PCS12 groups displayed a significantly higher mitochondria respiration compared to that in the Ctrl group (*P* < .05). Because OCR alternations in PCS6 and PCS12 groups were similar, PCS12 group was removed in the following experiments. To evaluate whether high mitochondrial respiration was sustained after prolonged PCS treatment, capacity of mitochondrial respiration in H9C2 cells was determined after four‐week PCS treatment. By comparing to vehicle control, PCS6 group still had significant improvements in the parameters of mitochondrial respiration, including basal respiration (*P* = .0053), maximal respiration (*P* < .0001), ATP production (*P* = .0129) and spare respiratory capacity (*P* = .0008) (Figure 2C,D).

### 3.3 PCS‐induced alterations of mitochondrial biogenesis

**Figure 3:**
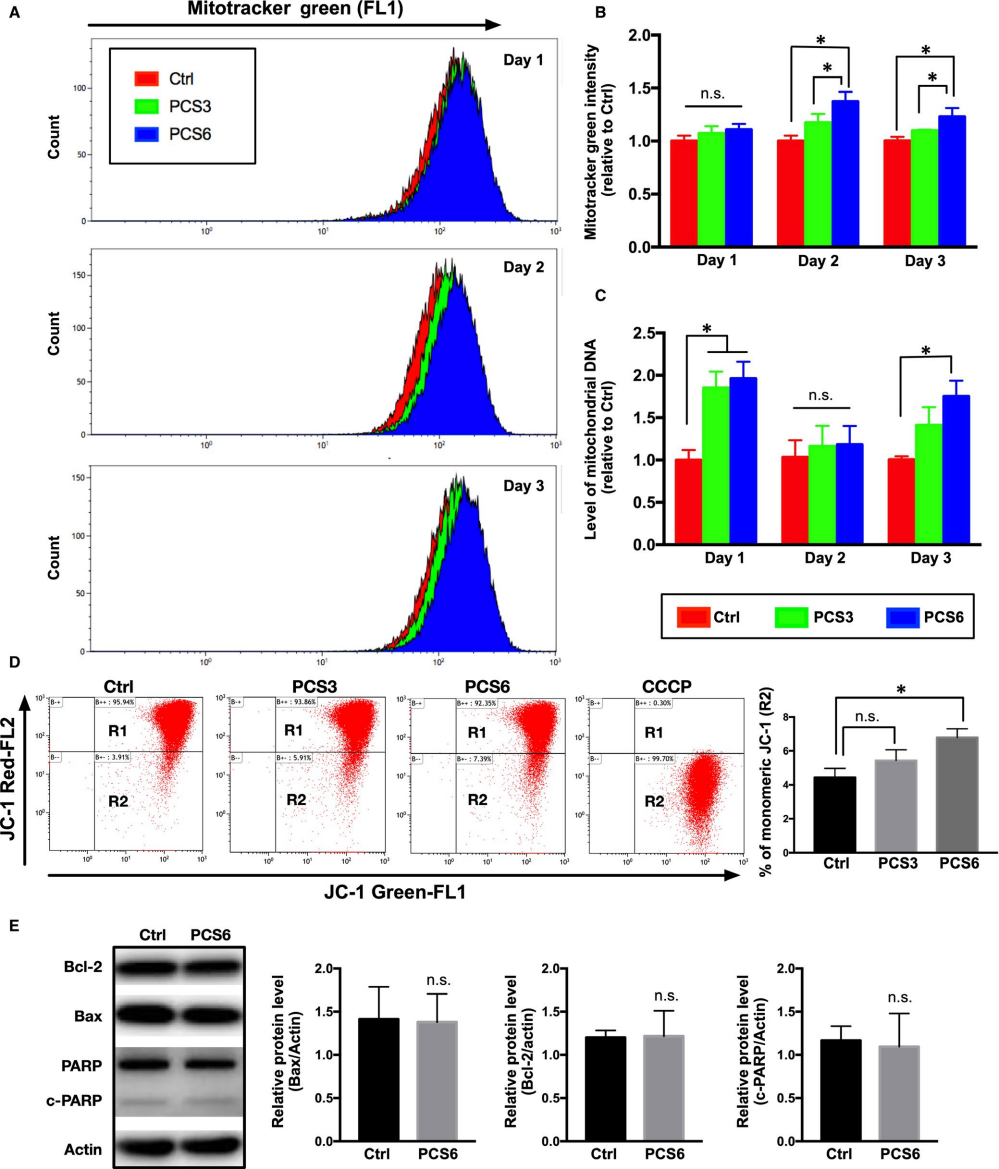
Changes in mitochondrial mass and mitochondrial DNA after PCS treatment. (A) Representative plots of mitochondrial mass in FACS analysis after mitotracker green staining (n = 3 per group) on Day 1, Day 2 and Day 3 following PCS treatment. (B) Fluorescence intensity of mitotracker green normalized by control (Ctrl) group (n = 3 per group). (C) Levels of mitochondrial DNA by quantitative PCR (n = 3 per group) on Day 1, Day 2 and Day 3 after PCS treatment. (D) Depolarization of mitochondrial membrane potential (ΔΨm) on FACS analysis of JC‐1 mitochondrial potential dye through representative dot plot. Gated region R1 (upper right) included cells with intact mitochondrial membranes, and gated region R2 (bottom right) included cells with loss of mitochondrial membrane potential. Treatment of CCCP caused mitochondrial membrane depolarization, as positive control. Percentage of monomeric JC‐1 (R2) revealed the state of ΔΨm after PCS treatment (n = 3 per group). (E) For the apoptosis evaluation, total cell lysates subjected to Western blot analysis with the indicated apoptosis markers including Bcl‐2, Bax and c‐PARP. Actin used as a loading control. Histograms are showing the relative densities. Each error bar represents the mean with SD, *P < .05, and n.s. indicates not significant

To estimate the effect of PCS treatment on mitochondrial biogenesis, mitotracker green staining and quantitative PCR were applied in Ctrl, PCS3 and PCS6 groups for the measurements of mitochondrial mass and mitochondrial DNA, respectively. After PCS treatment, the amount of mitochondrial mass in PCS3 and PCS6 groups was significantly increased on Day 2 and Day 3 compared to that in Ctrl group (Figure 3A,B). Furthermore, increased mitochondrial DNA copy number was also noted in PCS3 and PCS6 groups on Day 1 and Day 3 (Figure 3C). To estimate the impairment of mitochondria reflected by loss of mitochondrial membrane potential (ΔΨm) in response to PCS treatment, FACS analysis was performed using JC‐1 staining (Figure 3D). Comparing to that in the Ctrl group, PCS6 group displayed a significantly small increase in monomeric JC‐1 expression (*P* < .05) that indicated a slight loss of ΔΨm. Only 2.36% increase in monomeric JC‐1 expression on average was revealed in H9C2 cells after PCS (6.25 µg/mL) treatment. To determine whether H9C2 cardiomyoblasts displayed cellular apoptosis in response to PCS treatment, apoptotic protein expression in PCS6 group was determined by Western blot after three days of PCS treatment (Figure 3E). PCS6 group maintained low expressions of Bcl‐2, Bax and cleaved fragment of poly‐ADP‐ribose polymerase (PARP) as compared with those in Ctrl group, suggesting that treatment with the lowest effective dose of PCS (6.25 µg/mL) did not trigger apoptosis in H9C2 cardiomyoblasts.

### 3.4 PCS‐induced mitochondrial hyperfusion mediated by AMPK

**Figure 4:**
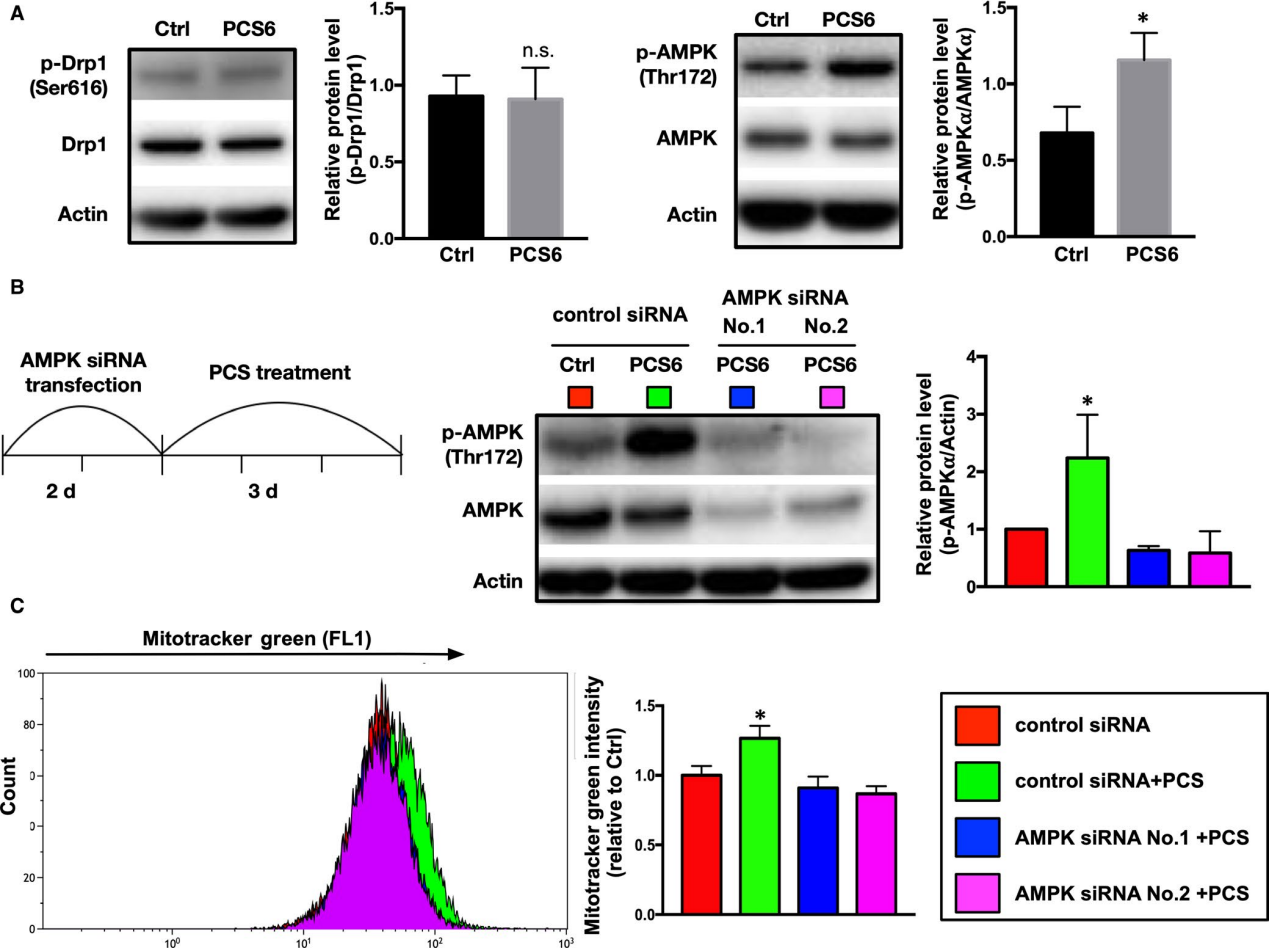
Importance of adenosine monophosphate‐activated protein kinase (AMPK) in PCS‐induced mitochondrial hyperfusion. (A) H9C2 cardiomyoblasts after three days of PCS treatment. Total cell lysates subjected to Western blot analysis with the indicated antibodies. Actin used as a loading control. Histograms are showing the relative densities of phosphorylated Drp1 (Ser616) versus total Drp1 and phosphorylated AMPK (Thr172) versus total AMPK (n = 3 per group). (B) Timeline of specific AMPK small interfering RNA (siRNA) transfection and PCS treatment in H9C2 cardiomyoblasts. Protein expression of AMPK after two different siRNAs transfection, evaluated by Western blots. (C) Representative plots of mitochondrial mass in FACS analysis after mitotracker green staining in groups of control siRNA, control siRNA combined PCS (6.25 µg/mL) treatment (control siRNA + PCS), and two different AMPK siRNAs (No.1 & No.2) combined PCS (6.25 µg/mL) treatment (AMPK siRNA + PCS). Fluorescence intensity of mitotracker green normalized by control group (Ctrl) (n = 6 in each group). Each error bar represents the mean with SD, *P < .05

Both dynamin‐related protein‐1 (Drp1) and adenosine monophosphate‐activated protein kinase (AMPK) mediate the process of mitochondrial fission in response to stress.^17^, ^18^ To determine whether both Drp1 and AMPK were associated with PCS‐induced mitochondrial hyperfusion, protein expression and phosphorylation of Drp1 and AMPK were examined (Figure 4A). After three days of PCS treatment, we found that AMPK phosphorylation (Thr172) was increased in PCS6 group compared to those in Ctrl group (*P* < .5) but Drp1 phosphorylation (S616) did not show significant difference. To clarify the importance of AMPK involved in PCS‐induced mitochondrial hyperfusion, knockdown of AMPK protein expression was conducted by siRNA transfection. After two days of AMPK siRNA transfection with H9C2 cardiomyoblasts, treatment of low concentration PCS (6.25 µg/mL) for three days was continuously performed (Figure 4B). We observed that the decrease of PCS‐induced mitochondrial hyperfusion regarding to the recovery of mitochondrial mass (Figure 4C) was demonstrated in AMPK‐silenced H9C2 cardiomyoblasts. Therefore, we assumed that AMPK protein expression plays an important role in PCS‐induced mitochondrial hyperfusion.

### 3.5 PCS‐induced cell enlargement

**Figure 5:**
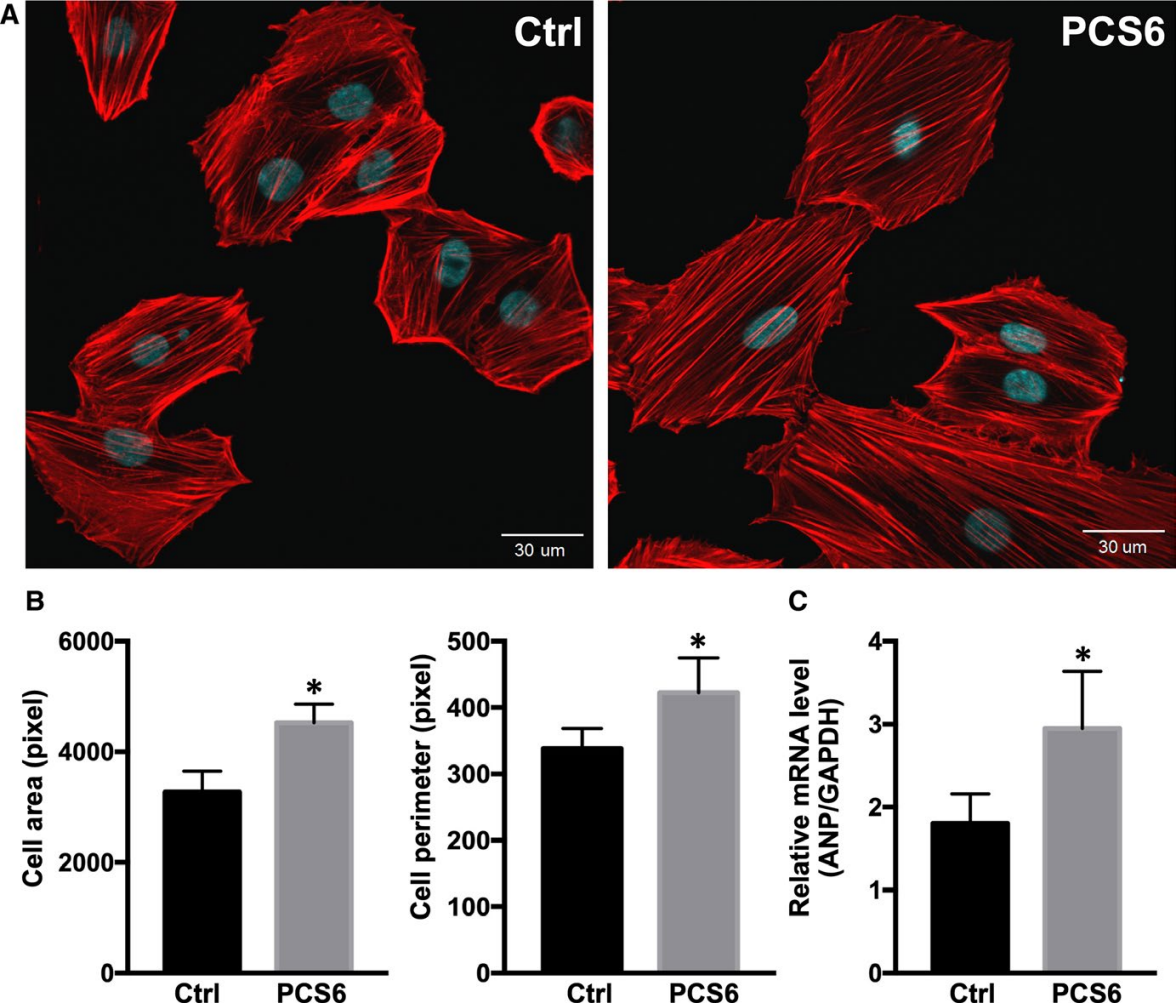
Measurement of cell size and quantification of hypertrophic gene expression after PCS treatment. (A) Vehicle control (Ctrl) with DMSO only and 6.25 µg/mL PCS (PCS6) applied in H9C2 cardiomyoblasts for the alternation of cell size on Day 21 after treatment. Representative morphologies of H9C2 cardiomyoblasts after mitotracker orange staining (displayed in red) and DAPI staining (displayed in blue). (B) Measurement of cell area and cell perimeter using ImageJ software. (C) Expression of atrial natriuretic peptide (ANP, myocardial hypertrophy‐related gene) determined by RT‐qPCR. Each error bar represents the mean with SD, *P < .05, and px indicates pixel

To assess the cellular alternation in hypertrophy after PCS treatment, the appearance of treated H9C2 cardiomyoblasts was quantitated for the cellular morphologic parameters such as cell area and perimeter. After three weeks of PCS treatment, both cell area and parameter in PCS6 group were higher than those in Ctrl group (*P* < .05) (Figures 4B, 5A). Furthermore, myocardial hypertrophy‐related gene expression, atrial natriuretic peptide (ANP) was also determined. We found that higher ANP mRNA expression in PCS6 group was revealed compared to Ctrl group (*P* < .05) (Figure 5C), suggesting that hypertrophic responses should be noted after PCS treatment.

### 3.6 Mitochondrial hyperfusion reversed after cessation of PCS treatment

**Figure 6:**
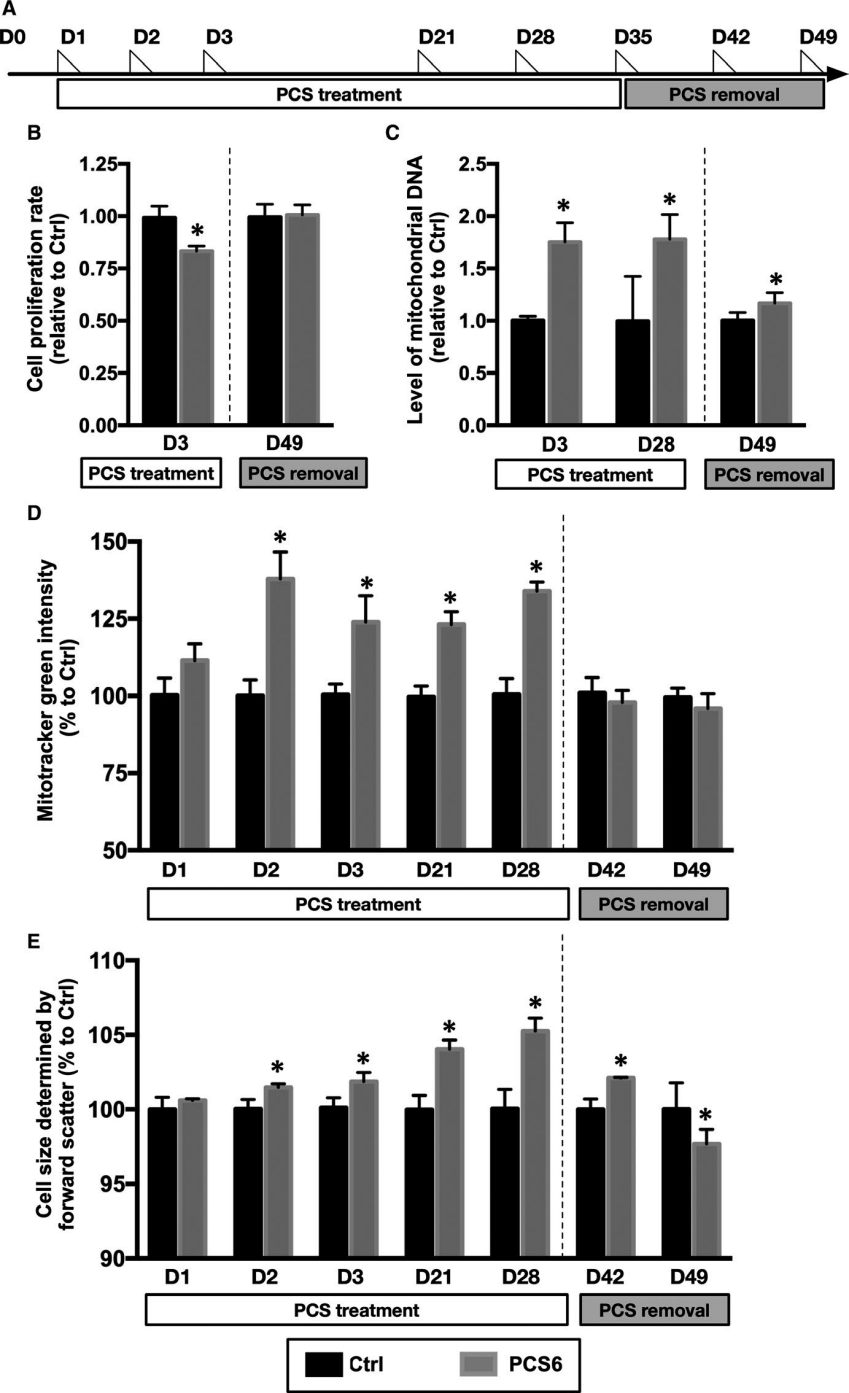
Changes in mitochondrial mass, mitochondrial DNA and cell size before and after PCS removal. (A) Timeline for PCS treatment (6.25 µg/mL, blank box) and cessation of PCS treatment (grey box). H9C2 cells collected at different days for assessing the changes in mitochondrial mass, mitochondrial DNA and cell size. The day of cell collection marked by triangle (D1 = Day 1). (B) Cell proliferation rates in Ctrl (black column, n = 8) and PCS6 (grey column, n = 8) groups using CCK8 assay. (C) Levels of mitochondrial DNA by quantitative PCR (n = 3‐5 per group). (D) Levels of mitochondrial mass assessed using FACS analysis of mitotracker green staining (n = 3‐6 per group). (E) Intensity of forward scatter in FACS analysis for evaluating changes in cell size with the dotted line indicating the presence or absence of PCS. Each error bar represents the mean with SD, *P < .05

To verify the degree of mitochondrial recovery after cessation of PCS treatment, we implemented a five‐week PCS treatment before cessation of PCS for two weeks (Figure 6A). After PCS cessation for two weeks, cell proliferation rate returned from 83% to 100% (Figure 6B). Also, cellular mitochondrial content reversed from 175% to 117% (Figure 6C). During the period of PCS treatment, persistently high mitochondria mass compared with that in the control group was noted from Day 2 to Day 28, and the mitochondrial mass returned to normal one week after cessation of PCS treatment (Figure 6D). After four weeks of PCS treatment, PCS‐treated H9C2 cells displayed an average 105% increase in cell size compared to that in the controls, and the increase was reversed in the absence of PCS (Figure 6E). Taken together, PCS‐induced increment in mitochondrial mass was associated with an increase in cell size, whereas mitochondrial hyperfusion was reversed after elimination of PCS stimulus in H9C2 cardiomyoblasts.

## 4 DISCUSSION

The major findings of this study were that low concentration of PCS not only morphologically caused mitochondrial hyperfusion but also functionally improved mitochondrial respiration in H9C2 cardiomyoblasts. We successfully established a PCS‐induced mitochondrial hyperfusion model for short‐term (3 days) and long‐term (4 weeks) studies. According to our results, the responses of short‐term PCS treatment were similar to those of long‐term PCS treatment, including the changes in mitochondrial DNA copy number, mitochondrial mass and mitochondrial respiration. This is the first study reporting that exposure of H9C2 cardiomyoblasts to mild PCS could enhance mitochondrial performance through the up‐regulation of biogenesis and bioenergetics.

Stress‐induced mitochondrial hyperfusion (SIMH) response is thought to be an adaptive pro‐survival response against stress.^10^ Our findings demonstrated that PCS is a novel stress stimulus that could trigger SIMH in H9C2 cardiomyoblasts. Total serum/plasma PCS concentrations in normal subjects have been reported to vary in range from 2.8 ± 1.7 µg/mL^19^ to 6.6 ± 3.7 µg/mL,^20^ determined by different methods, respectively. After renal injury, the mean plasma concentration of PCS accumulated in patients with end‐stage renal disease is up to 106.9 ± 44.6 µg/mL.^21^ High concentrations of PCS, varying from 28 to 94 µg/mL, have been reported to induce oxidative stress in various cells in vitro, such as human umbilical vein endothelial cells (HUVECs), human vascular smooth muscle cells (HVSMC), human renal proximal tubule cells (HK‐2) and H9C2 cardiomyoblasts.^4^, ^5^, ^6^ High levels of oxidative stress that lead to cell apoptosis also cause excessive mitochondrial fission.^22^ In this study, we have shown that low concentrations of PCS only caused slight mitochondrial damage without triggering cell apoptosis. Hyperfused mitochondria accompanied by increased mitochondrial respiration was observed under low PCS concentration (6.25 µg/mL), implying that SIMH may be a protective response against PCS insults. The findings of the current study suggested that PCS may induce an apparent energy imbalance that triggers AMPK activation which, in turn, causes mitochondrial hyperfusion.

SIMH is a compensatory response through mitophagy suppression^23^ in response to external stress that mandates increased cellular energy (ie ATP) production.^10^ If the ATP demand is not promptly satisfied, it will lead to initiation of senescence or induction of apoptosis.^24^ Our findings indicate that increases of mitochondrial mass and mitochondrial DNA copy number after PCS treatment may allow physiological elongation and interlacing between mitochondria. Consequently, hyperfused structures may meet the sudden increase in energy demand through producing extra amount of ATP through oxidative phosphorylation.

Recent evidence also suggests that hyperfused mitochondrial networks suppress cell proliferation through the interruption of cell division.^25^ In our study, treatment of PCS with low concentration did not cause cell apoptosis. Therefore, we speculate that reduced cell number following consecutive PCS treatment may be caused by cell cycle arrest rather than apoptosis. On the other hand, AMPK not only maintains metabolic homoeostasis through the regulation of mitochondrial dynamics^18^ but also plays a crucial role in mitochondrial biogenesis.^26^ Consistently, our findings indicate that increased AMPK activity may be associated with hyperfused mitochondria and irregular mitochondrial metabolism following PCS treatment. Besides, the results of the current study suggested that mitochondria may not only control the cellular growth rate but also determine the cell size. Because overcrowded organelles (eg mitochondria) and cytosolic particles could slow down the rate of intracellular diffusive transport for oxygen and nutrients, there may be a compensatory increase in cell size to facilitate intracellular diffusion.^27^, ^28^ Additionally, Myers et al have demonstrated that pan‐AMPK activator not only enhances glucose uptake but also causes cardiac hypertrophy.^29^ With regard to the control of mitochondrial networks, Drp1 is a critical mediator in mitochondrial dynamics. Loss of Drp1 impairs mitochondrial fission and leads to the formation of tubular morphology composed by elongated mitochondria.^22^, ^30^ However, Drp1 expression and activity in response to PCS treatment did not display significant differences. We assume that PCS‐induced mitochondrial hyperfusion may be mainly regulated by AMPK rather than Drp1‐mediated mitochondrial dynamics.

It has been reported that H9C2 cardiomyoblasts and primary neonatal cardiomyocytes could display similar hypertrophic responses in vitro.^31^ Expression of ANP has been reported to be closely associated with the progression of myocardial hypertrophy^32^ and is considered to play an protective role in compensation.^33^ Markedly elevation of ANP expression in heart tissue has been commonly used as a hypertrophic marker.^34^ During long‐term PCS treatment, we observed an association between an increase of mitochondrial mass and cell enlargement. Notably, cell size returned to normal after cessation of PCS treatment. This response is similar to physical exercise‐induced physiological cardiac hypertrophy in response to the increased workload. This increase in cell size cannot be sustained unless the exercise is maintained.^35^ Unlike physiological cardiac hypertrophy, pathological cardiac hypertrophy induced by disease conditions is less reversible.^36^ Therefore, because low concentration PCS‐induced cell hypertrophy was reversible in the present study, we assume that it was more likely a physiological rather than a pathological response.

Recently, it has been reported that metformin is able to raise the survival rate in older patients with moderate CKD.^37^ Metformin is widely used as an antiglycemic drug to treat diabetes. Beyond the beneficial outcome of glucose reduction, administration of metformin may extend lifespan through the release of a small amount of reactive oxygen species (ROS) from mitochondria to trigger a cellular process against oxidative stress.^38^ Furthermore, metformin may be able to lead the protection of myocardium and preservation of heart function through the activation of AMPK signalling pathway.^39^ We speculate that low‐level PCS in CKD may act as a kind of cellular stimuli, such as metformin, which may activate AMPK signal and generate excess ROS through mitochondrial hyperfusion.

The present study has its limitations. Despite the findings of the effects of low concentration PCS on mitochondrial morphology and function, the underlying mechanism of mitochondrial hyperfusion and its after‐effect on homoeostasis remains unclear. Besides, although significant changes in mitochondrial mass and corresponding cell size have been demonstrated, the relationship may be further elucidated with more precise measurement, such as transmission electron microscopy. Moreover, the current study only evaluated the changes in mitochondrial respiration using the energy from glucose in culture medium without assessing the regulation of cellular energetic efficiency in the utilization of different fuels (ie protein and fatty acid) under the condition of mitochondrial hyperfusion. The discovery of PCS‐induced mitochondrial hyperfusion in this study may open new avenues to understanding the cellular rescue strategies against stress.

In conclusion, the results of the current study demonstrated that low‐level p‐cresyl sulphate (PCS) caused AMPK‐dependent mitochondrial hyperfusion in H9C2 cardiomyoblasts with increased mitochondrial biogenesis, enhanced mitochondrial respiration and enlarged cell size.

## CONFLICTS OF INTEREST

The authors report no relationships that could be construed as a conflict of interest.

## **AUTHOR** **CONTRIBUTION**

THH and YLC conceived the study and participated in the design of the study, data acquisition and analysis, and drafting the manuscript. YLC and THH were responsible for the laboratory assay and troubleshooting. HKY, CKS, CCY and FYL participated in interpretation. All authors read and approved the final manuscript.

## Data Availability

The data that support the findings of this study are available from the corresponding author upon reasonable request.
